# Testing a nutrient composition threshold model to classify brands for marketing restrictions

**DOI:** 10.1371/journal.pone.0311579

**Published:** 2024-10-25

**Authors:** Rachel Jordan, Kelly Garton, Sally Mackay

**Affiliations:** Faculty of Medical and Health Sciences, School of Population Health, The University of Auckland, Auckland, New Zealand; Oswaldo Cruz Foundation: Fundacao Oswaldo Cruz, BRAZIL

## Abstract

**Introduction:**

Food marketing restrictions often apply nutrient profile models (NPM) to distinguish unhealthy products that should not be advertised, however brand-only marketing remains largely unaddressed. We sought to test a threshold method for classifying packaged food, beverage, or fast-food brands as (non)permitted for marketing, based on the nutrient profile of their product-lines.

**Methods:**

We retrieved nutrient information from the Nutritrack databases for all products sold by the top 51 packaged food, beverage and fast-food brands in New Zealand, selected by market share. All products under each brand were classified as permitted (or not) to be marketed to children, using the NPM for WHO Western Pacific. The 25%, 50%, 75% and 90% threshold of brands’ products permitted to market were compared. The 50% and 75% thresholds were compared to the WHO CLICK method, which is based on assessment of the brand’s leading product.

**Results:**

The 90% threshold permitted 13% of the brands to be marketed to children. The 25% threshold permitted the marketing of 62% of brands. The 50% and 75% thresholds remained highly sensitive in identifying brands that should not be marketed to children. Comparison to the WHO CLICK method identified that a threshold method is more comprehensive and less arbitrary.

**Conclusions:**

A threshold model based on product-line nutrient profiling provides a robust and option for brand classification. The 50% and 75% thresholds may be the most politically preferred options for use in regulation, while remaining highly effective.

**Practitioner points:**

- Brand marketing (e.g. sponsorship) remains largely unaddressed in existing restrictions on unhealthy food and beverage marketing to children.
- An established Nutrient Profile Model can be applied to a brand’s entire product line, allowing calculation of the proportion of products that would not be permitted to be advertised to children.
- Restricting brand marketing for food and beverage brands with less than 50% or 75% of their products classified as ’permitted to be marketed to children’ is a robust and evidence-based method that can be applied in regulation, with potential to mitigate industry challenges.

## Introduction

Childhood nutrition sets a foundation for health and development throughout the life course. The WHO-UNICEF-Lancet Commission identified that the ongoing exploitation of children through marketing practices, particularly for unhealthy food and beverages, is a key threat to health [1]. Children are exposed to harmful marketing through various mediums, and the rise of digital marketing has significantly increased the potential for children to be exposed given ubiquity of digital media, and challenges in monitoring exposure [2–13]. Unhealthy product marketing targets children because of the opportunity to influence their developing preferences, taking advantage of their inability to discern between truth and persuasion [14]. In addition to the immediate effect of marketing on children’s consumption behaviours and family spending, brand loyalty built during childhood is likely to be transferred through to their adult years as lifelong consumers [15]. The lasting impact on eating behaviours and preferences has been clearly linked to an increasing burden of excess weight and non-communicable disease from childhood [14,16,17]. Given these findings, the World Health Organization (WHO) issued a set of recommendations to reduce marketing of unhealthy foods and beverages to children in 2010 and 2016 [14,18]. Despite unanimous support from the World Health Assembly, implementation of the WHO recommendations since 2010 has been limited [18].

Policy recommendations from the WHO include using government-led nutrient profile models to classify foods that should be restricted, and that children of all ages (up to age 18) should be protected by policy [19]. Policy also needs to be comprehensive enough to ensure that brands do not migrate marketing to other media and can sufficiently restrict the persuasive power of food marketing [19]. The policy recommendations also note the risk of brands increasing brand-only marketing if restrictions are not sufficiently comprehensive [19].

Additionally, the WHO recommends that jurisdictions implement comprehensive mandatory restrictions given that self-regulatory codes have consistently failed to protect children [11,14,18,19]. Enforcement functions for rules around marketing should be allocated to the Ministry of Health or an equivalent health-related agency [19,20]. Increasing use of targeted digital media highlights the need for comprehensive action that includes governance of the digital marketing landscape [20].

There are currently no restrictions, voluntary or otherwise, on what brands can sponsor or disseminate brand-only marketing in New Zealand (NZ). Recent research on the nature of food and beverage marketing in NZ indicates that brand-only marketing is on the rise [21]. Children are likely to associate certain brands with unhealthy food, and exposure to brand sponsorship may increase their intention to consume and build long-term brand loyalty for such products [22]. Sponsorship is another tool used by brands to build children’s awareness and preferences [23]. An analysis of popular sports clubs in NZ was completed in 2019/2020 showing that 28% of clubs were sponsored by a food or non-alcoholic beverage brand [2]. Fast-food chains made up 21% of the sponsorships [2]. Sponsorship is therefore an effective way for young people to be exposed to brand marketing and may be particularly effective given the connection children can hold with their sports clubs [6].

Despite well-established methods for classifying individual products, there is relatively little focus on methods for classifying brands. The WHO has developed a method for identifying if a brand is healthy or unhealthy as part of the CLICK framework. In this method, the top-selling or most visible product of a brand is identified and assessed by a reviewer to be healthy or unhealthy, and this label is applied to the brand as a whole [15]. While this is a relatively simple available method, it lacks rigour and may be open to industry challenge given that it may be perceived as highly ‘arbitrary’ [24]. A variety of other ad hoc methods have been utilised to classify brands in studies monitoring food marketing [6–8,12,25–35]. However, there has yet to be established a rigorous, evidence-based, user-friendly and internationally-recognised method to classify brands for the purpose of marketing restrictions.

To address this gap in NZ policy and international best practice, a quantitative exploratory study was completed to assess the implications of nutrient profiling with various potential thresholds for classifying a brand as ‘healthy’ or ‘unhealthy’, i.e. permitted to be marketed or not. The primary aim of the study was to analyse how varying thresholds would impact whether/how many of the top brands by market share in NZ would be permitted to market to children. A secondary aim was to compare the effect of thresholds to the WHO CLICK method for classifying a brand.

## Methods

This study utilised the Nutritrack database to nutritionally assess products from packaged food and beverages and fast food chain brands. Nutritrack is owned and managed by the National Institute of Health Innovation at the University of Auckland. The Nutritrack packaged foods database is updated annually by trained researchers with information regarding the nutrient composition of packaged foods and non-alcoholic beverages sold at four major national supermarkets in Auckland, NZ (New World, Countdown, Four Square, and Pak’N Save) [36]. The Nutritrack fast foods database is an annual cross-sectional survey of all food and beverage products available for sale at fast-food chains in NZ with twenty or more stores. Data on product names, serving, pack sizes and nutrient information is collected by trained researchers from company websites and in-store for the database.

**Fig 1:**
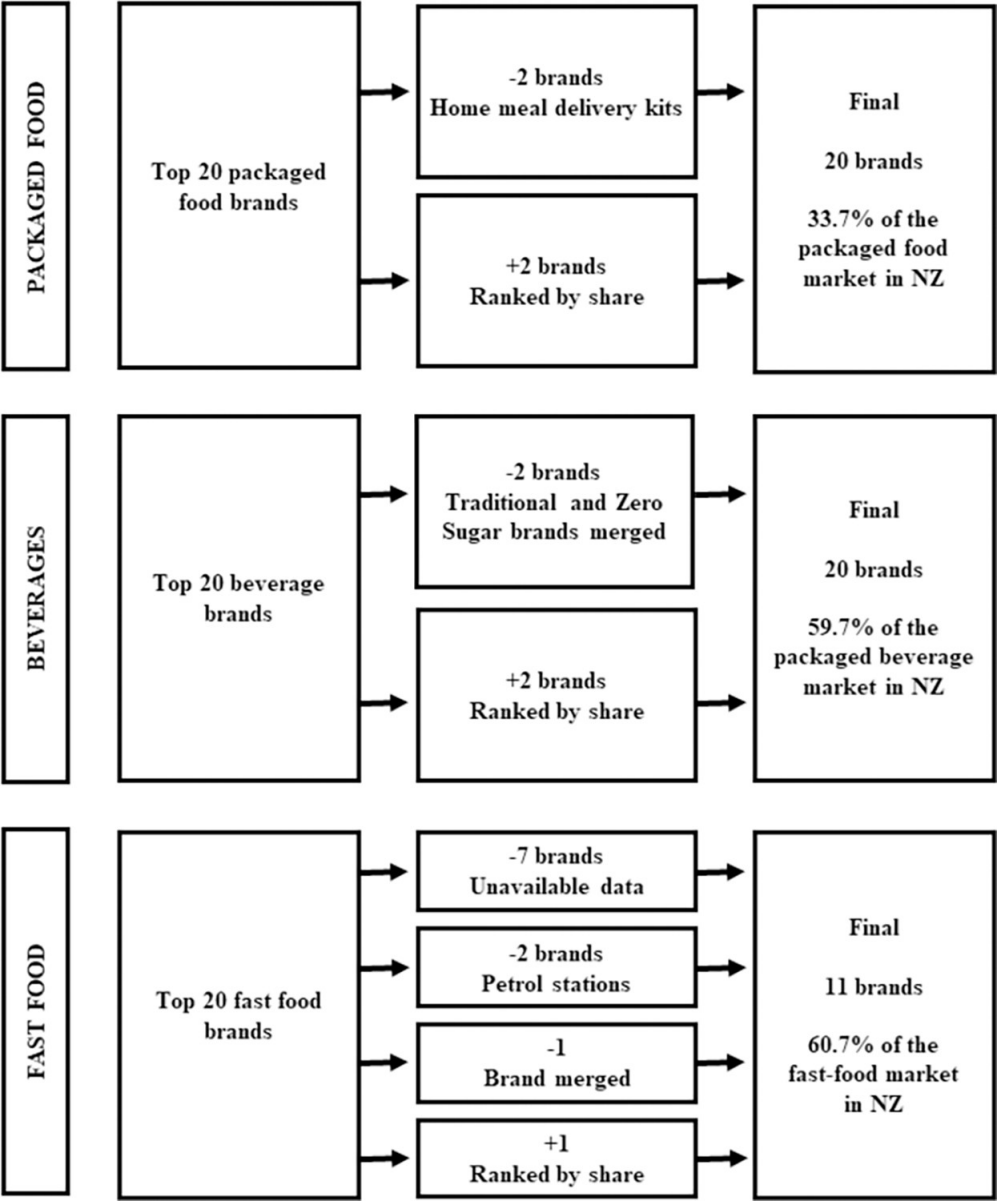
Process to select packaged food, beverage and fast-food brands for analysis.

Brand names were the focus of the analysis rather than the global parent company as advertisements that children will be exposed to are typically associated with the brand name. The process for selecting the top packaged food, beverage and fast-food brands is outlined in Fig 1. The top 20 packaged food brands by brand shares in 2021 in NZ were identified through the Euromonitor Passport portal [37]. Two brands that sold home meal delivery kits, My Food Bag and Hello Fresh, were excluded from the analysis; hence, the next two packaged food brands ranked by brand share were added to the analysis sample to have twenty brands in total. Three brands included in the packaged food section of Euromonitor also sold beverages under this brand name (Anchor, Mainland, and Sanitarium). These beverages were included under the packaged food brand category, as they would be recognised as one brand for advertising purposes. The selected brands represented 33.7% of the packaged food market in NZ in 2021 [37]. The same process was used to identify the top 20 beverage brands. Coca-Cola and Coca-Cola Zero Sugar were classified as one brand, as were Sprite and Sprite Zero. This was done as the Nutritrack database categorises them as one brand and the research team considered they would likely be commonly identified as an inclusive brand. Again, the next two beverage brands by brand share were then added to the sample for analysis. The selected brands represented 59.7% of the packaged beverage market in NZ in 2021 [37].

The top 20 fast-food brands were also identified by Euromonitor data. Six of these brands could not be assessed; their nutrition data was not collected in the Nutritrack 2020 database for fast foods as it was not publicly available. The brands excluded were Noodle Canteen, Lone Star, Columbus Coffee, The Coffee Club, Mobil, and Nando’s. Two petrol stations that serve food, Z petrol station café and BP, were also excluded from the analysis. McDonald’s and McCafé were considered one brand under McDonald’s as their products are all served in a McDonald’s premises and Nutritrack classifies all the products under the McDonald’s brand name. St Pierre’s Sushi was added to the analysis as the next available fast-food brand, however the next largest brands did not have nutrient data available on Nutritrack. Overall, twelve fast food brands in total were selected as suitable for analysis. During analysis, one fast-food brand (Pita Pit) was excluded due to a high proportion of products missing nutrient data, bringing the final total to 11 fast-food brands. The remaining eleven brands selected represented 60.7% of the fast-food market share in NZ in 2020 [37].

The required nutrient information was transferred from the relevant Nutritrack datasets to an excel spreadsheet in January 2022 for each product sold under the selected brands. Ingredient lists were also included for the packaged food and beverage brands to enable assessment of added sugars. Ingredient lists were not included in the fast-food dataset as this information is often not available. The nutrient profile model (NPM) of the WHO Western Pacific Regional Office (WPRO) was then manually applied by hand to each product to categorise as permitted or not permitted to be marketed to children and entered into Excel. This model was created specifically for countries in the Western Pacific region; it was selected as it is most relevant to the NZ food setting and aligns with the WHO recommendations for what foods should and should not be marketed to children, i.e., the model specifically categorises food and beverage products as either permitted or not permitted to be marketed to children. The appropriate category was selected for each product by using the descriptors provided in the nutrient information field. For most products, it was straightforward to select the category. Any products that were unclear or could potentially fit in two categories were discussed with the senior author, who is experienced in using the model, and a consensus was reached. For packaged foods and beverages, a product was not assessed against the NPM if the nutrient data was unavailable in the dataset. It was not feasible within the time constraints of this study to follow up with individual brands to obtain missing nutrient data, however this could be an option for the future. Regardless, only 14 packaged food and 0 beverage products in the database were missing data. In the fast-food brand analysis, all beverage products sold at an outlet were included, i.e., branded varieties such as Coca-Cola’s product line. None of the beverages available at McCafé had nutrient data available. As a large proportion of the products listed under McCafé were coffee-based beverages (70%), they would not be permitted in the NPM anyway, so these were coded as such. Ingredient lists were assessed online on the McDonald’s website for other beverages when available.

Once all products (packaged food and beverage, and fast food/beverage) had been classified, the percentage of each brand’s product-line that would be permitted to be marketed to children was calculated. The following thresholds were selected to test for whether brands would be permitted to market to children: 25%, 50%, 75% and 90% using STATA.

The WHO CLICK method categorises a brand by selecting either the top-selling product from sales data or the product that is first visible on the brand website if sales data is unavailable [16]. The method was established in 2019 and was published by the WHO Office for Europe. The overarching monitoring framework was created through workshops with public health experts [16]. Following the analysis of thresholds, the WHO CLICK method was applied to each of the sampled packaged food and beverage brands. Fast food brands were not assessed as most of the identified leading products were unavailable in the Nutritrack database for fast foods so accurate analysis using the WHO CLICK method would not be possible. As the sales data was not available for each brand, the website method for the first visible product was used. Once a product was selected, it was categorized as permitted or not permitted using the WHO WPRO NPM. Three brands (McCain, Sealord, and Tegel) did not have a clearly visible product on the first page. In this instance the first product visible on the product page of each website was selected for analysis. Three selected brand products (McCain, Fresh’n Fruity and Powerade) were not available on the Nutritrack database used for the analysis, however nutrient information was present on the website for classification of the product. This is possibly because the 2019 database was used for nutrient information, whereas websites hold current product lines which may have changed since collection of the database. Each brand’s classification for the WHO method was then compared to the 50% and 75% threshold results for the brand.

## Results

1550 food products, 327 beverages and 1333 fast food products were classified using the WHO WPRO NPM. Fourteen packaged food products and zero beverage products were unable to be classified due to lack of nutrient information available in the dataset. The packaged food products with data missing were sold under a range of brands so they were removed from the dataset with no adjustments made to the analysis. The fast-food dataset had a large number of products without nutrient information available (n = 1827, 58% of products). The number of products with missing information varied significantly between each fast-food brand. Burger King had the most products with nutrient data available (90%) while Pita Pit had the least (13%). In some circumstances, information was available for one variation of a product, e.g. Subway providing nutrient data for a white bread option of a meal but omitting full nutrient data for other bread options listed on the dataset. However, many products that could be classed as a product variation did not have any nutrient information available for any of the variations.

The percentage of products permitted to be marketed varied by brand type. Drinks brands had the lowest percentage with 7% permitted to be marketed, while packaged foods had the highest at 37%. Table 1 shows the overall proportion of products permitted and not permitted under each category of brand, and more detailed tabulation of products analysed and classified as permitted (or not) to be marketed–separated by packaged food, beverage, and fast food brand–can be found in S1–S3 Tables, respectively. Of the packaged food brands, eight had no products sold that would be permitted to be marketed to children. This usually reflected the brand category as being synonymous with being ‘unhealthy’ such as confectionary, biscuits, savoury snack foods and ice cream. For example, Cadbury sells chocolate exclusively, a product which is automatically not allowed to be marketed to children under the WHO WPRO NPM [38]. Mainland is a dairy brand that sells a variety of common household cheeses, such as Edam. The saturated fat content of the cheeses sold exceeded limits under the model, so none of them were permitted to be marketed to children.

**Table 1:** Overall percentage of products permitted and not permitted under each category of brands.

Category of brands	Number of products	Products successfully classified, (%)	Percentage of products permitted (%)	Percentage of products not permitted (%)
Packaged foods	1564	99	37	63
Beverages	327	100	7	93
Fast foods	3161	43	20	80

Anchor, Fresh’n Fruity and Meadow Fresh are all brands that sell dairy products. The percentage of permitted products ranged from 50–60% across these three brands. Products not permitted were primarily cheeses due to elevated saturated fat and yoghurts due to sugar content. Sanitarium is primarily a cereal brand, and also sells breakfast shakes (e.g., Up and Go), and plant-based milks. Only 23% of Sanitarium’s products were permitted to be marketed to children, largely due to sugar content exceeding the permitted threshold in cereals and the presence of added sugar for milks.

Fifteen of the selected twenty packaged beverage brands had less than 20% of products permitted to be marketed to children, with a majority of these having 0% permitted (n = 11). These beverage brands were all either sweetened soda, juice or energy drink brands. H2Go is an exception as a water brand. However, the only H2Go products available on the Nutritrack databases were all sweetened flavoured waters, therefore these were all not permitted. H2Go do sell plain water products but these were not included in the analysis as they were not on the 2019 Nutritrack database. Five beverage brands had 75% or more products permitted to be marketed, and these were all brands that sold water-based products. E2, Red Bull and V all sold exclusively energy drinks, which are automatically not permitted to be marketed to children under the WHO WPRO model.

There was a range of proportions of products permitted to be marketed to children between the fast-food brands. Domino’s Pizza had the lowest proportion of products at 2% and St Pierre’s Sushi had the most at 70%. The brand category did not necessarily correlate with the proportion of products permitted. For example, pizza outlets Hell Pizza and Pizza Hut had 24% and 25% of products permitted respectively, compared to Domino’s which had just 2% of products permitted. Of note, Pita Pit had 17% of products permitted to be marketed. However, given the overall lack of nutrient data available for Pita Pit, the majority of products analysed were beverages and baked goods which does not reflect the overall product picture. Given this, Pita Pit was removed from the analysis, as noted previously.

### Threshold testing/assessment

**Fig 2:**
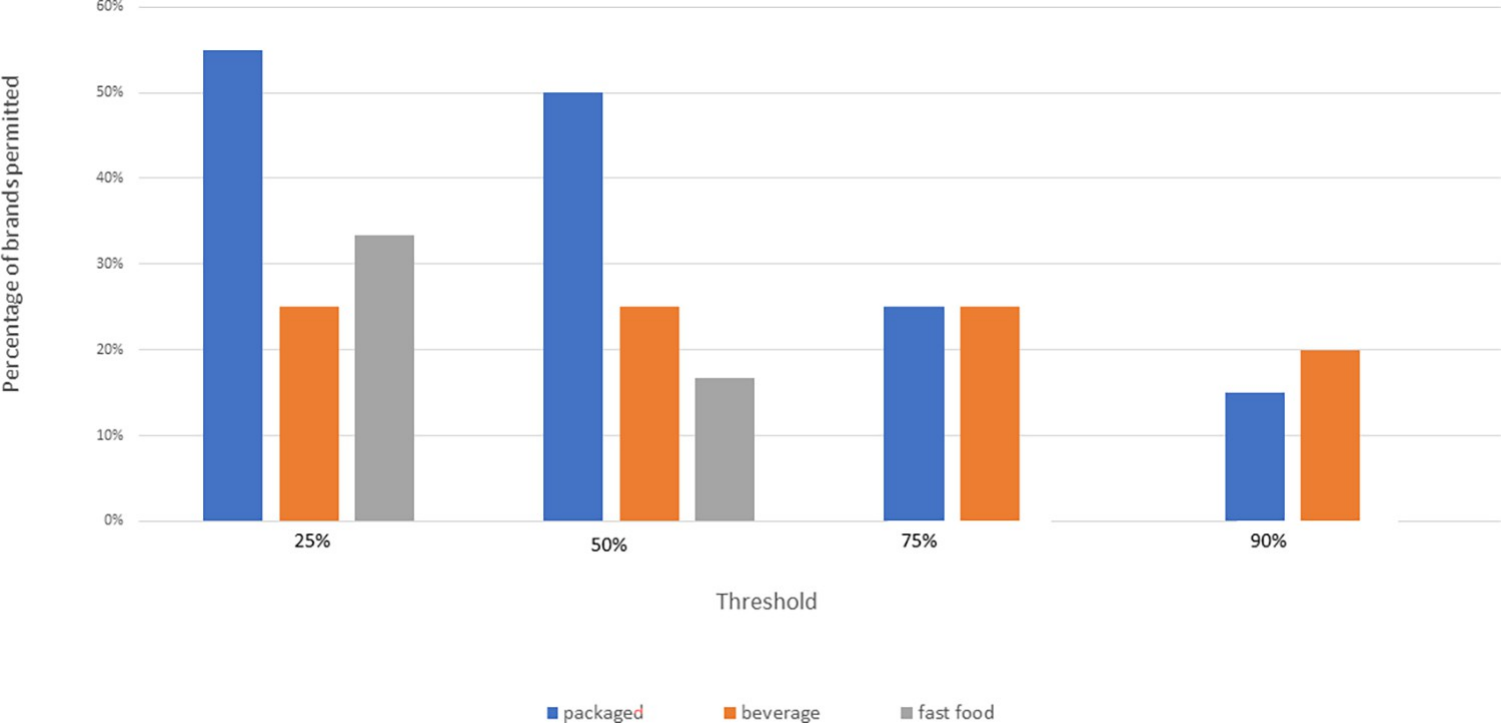
Percentage of brands permitted to market at each threshold. Packaged foods are represented in blue, beverages in orange, and fast-food in grey. Percentages were calculated from the total number of brands in each category.

The analysis compared which brands would and would not be permitted for marketing, when thresholds of 25%, 50%, 75% and 90% (percentage of ‘healthy’/permitted products) were applied. Fig 2 shows the percentage of brands permitted to market under each threshold.

The 90% threshold is the most restrictive option tested as part of this study. Applying a 90% threshold left three packaged food brands, four beverage brands and zero fast-food brands permitted to market to children. All beverage brands that would be permitted to market at this threshold were water-based brands. While some of the products sold were flavoured waters, none of these brands meeting the 90% threshold used non-sugar sweeteners or added sugar in their flavoured water formulations. Table 2 shows the brands permitted and not permitted to be marketed at the 90% threshold for packaged foods, beverages, and fast foods.

**Table 2:** Applying a threshold of brands permitted to be marketed.

	List of brands permitted to be marketed	List of brands not permitted to be marketed
*Applying a 90% threshold of brands permitted to be marketed*		
**Packaged foods**	Flora, Meadow Lea, Sealord	Anchor, Arnott’s, Bluebird, Cadbury, Eta, Fresh’n Fruity, Griffin’s, Maggi, Mainland, McCain, Meadow Fresh, Quality Bakers, Sanitarium, Tegel, Tip Top, Wattie’s, Whittaker’s
**Beverages**	Pure NZ, NZ Natural, Pump, Pure Dew	Charlie’s, Coca Cola, E2, Fresh Up, H2Go, Just Juice, Keri Juice Co, Kiwi Blue, L&P, Mizone, Powerade, Red Bull, Schweppes, Sprite, McCoy, V
**Fast-foods**		Burger Fuel, Burger King, Domino’s Pizza, Hell Pizza, KFC, McDonald’s, St Pierre’s Sushi, Subway, Pizza Hut, Tank Juice, Wendy’s
*Applying a 75% threshold of brands permitted to be marketed*		
**Packaged foods**	Flora, Meadow Lea, Quality Bakers, Sealord, Tegel	Anchor, Arnott’s, Bluebird, Cadbury, Eta, Fresh’n Fruity, Griffin’s, Maggi, Mainland, McCain, Meadow Fresh, Sanitarium, Tip Top, Wattie’s, Whittaker’s
**Beverages**	Kiwi Blue, Pure NZ, NZ Natural, Pump, Pure Dew	Charlie’s, Coca Cola, E2, Fresh Up, H2Go, Just Juice, Keri Juice Co, L&P, McCoy, Mizone, Powerade, Redbull, Schweppes, Sprite, V
**Fast-foods**		Burger Fuel, Burger King, Domino’s Pizza, Hell Pizza, KFC, McDonald’s, St Pierre’s Sushi, Subway, Pizza Hut, Tank Juice, Wendy’s
*Applying a 50% threshold of brands permitted to be marketed*		
**Packaged foods**	Anchor, Flora, Fresh’n Fruity, McCain, Meadow Fresh, Meadow Lea, Quality Bakers, Sealord, Tegel, Wattie’s	Arnott’s, Bluebird, Cadbury, Eta, Griffin’s, Maggi, Mainland, Sanitarium, Tip Top, Whittaker’s
**Beverages**	Kiwi Blue, Pure NZ, NZ Natural, Pump, Pure Dew	Charlie’s, Coca Cola, E2, Fresh Up, H2Go, Just Juice, Keri Juice Co, L&P, McCoy, Mizone, Powerade, Redbull, Schweppes, Sprite, V
**Fast-foods**	St Pierre’s Sushi, Subway	Burger Fuel, Burger King, Domino’s Pizza, Hell Pizza, KFC, McDonald’s, Pizza Hut, Tank Juice, Wendy’s
*Applying a 25% threshold of brands permitted to be marketed*		
**Packaged foods**	Anchor, Flora, Fresh’n Fruity, Maggi, McCain, Meadow Fresh, Meadow Lea, Quality Bakers, Sealord, Tegel, Wattie’s	Arnott’s, Bluebird, Cadbury, Eta, Griffin’s, Mainland, Sanitarium, Tip Top, Whittaker’s
**Beverages**	Kiwi Blue, Pure NZ, NZ Natural, Pump, Pure Dew	Charlie’s, Coca Cola, E2, Fresh Up, H2Go, Just Juice, Keri Juice Co, L&P, McCoy, Mizone, Powerade, Redbull, Schweppes, Sprite, V
**Fast-foods**	Pizza Hut, St Pierre’s Sushi, Subway, Tank Juice	Burger Fuel, Burger King, Domino’s Pizza, Hell Pizza, KFC, McDonald’s, Wendy’s

When the threshold is reduced to 75%, three further brands would be permitted to be marketed to children (Table 2). Tegel (primarily poultry products), Quality Bakers (breads), and Kiwi Blue (water and flavoured water) all met the 75% threshold. No further fast-food brands made this threshold.

A reduction to a 50% threshold would allow for five further packaged food brands to be marketed (Table 2). This notably included three dairy brands: Anchor, Fresh’n Fruity and Meadow Fresh. Anchor and Fresh’n Fruity both had 50% of products permitted to be marketed, while Meadow Fresh had 60%. High sugar contents in yoghurts and elevated saturated fats in cheeses were the main reasons for dairy brands having lower percentages permitted to be marketed to children. No further beverage brands were permitted to be marketed with this reduction. At the 50% threshold there were two fast-food brands permitted to be marketed: Subway and St Pierre’s Sushi.

Lowering the threshold further to 25% had minimal impact on the packaged food and beverage brands permitted to be marketed (Table 2). Even with this reasonably lenient threshold, the majority of fast-food, beverage and packaged food brands that would likely be considered as unhealthy were not permitted to be marketed. With a 25% threshold applied there was no change to the beverage brands permitted to market to children compared to the 75% and 50% thresholds. One further packaged food brand met this threshold: Maggi. Maggi sells a variety of products, mostly in the sauces and ready-made meals categories. Seven packaged food brands selected would never be permitted to be marketed to children as none of the products analysed through Nutritrack were permitted to be marketed to children. These packaged food brands largely reflect the confectionary and snack food categories. At 25%, two further fast-food brands could market: Tank Juice and Pizza Hut both met this threshold. Tank Juice primarily sells smoothies and fruit juices. The majority of the products available were classified as not permitted due to the sugar content being higher than the threshold allowed.

### Comparison to WHO CLICK method

Using the WHO CLICK method, the most visible product on the brand website was identified for each brand. A product was able to be identified and classified by the WHO WPRO NPM for all brands in the packaged food and beverage categories. Fresh’n Fruity and McCain both had ‘top’ products identified that were not available in Nutritrack, however the company websites included nutrient information for each product which allowed classification. Table 3 details the 50% and 75% threshold outcomes for each brand compared to the WHO CLICK method for packaged food brands. The 50% and 75% thresholds were chosen as the research team agreed following threshold testing that they would be the most feasible thresholds in practice.

**Table 3:** Comparison of WHO CLICK method to 50% and 75% thresholds for each packaged food brand.

Brand	Brand permitted at 50% threshold (Y/N)	Brand permitted at 75% threshold (Y/N)	Product selected with WHO CLICK method	Brand permitted using WHO CLICK method (Y/N)
**Anchor**	Y	N	Probiotic Plus Strawberry and Raspberry Yoghurt	Y
**Arnott’s**	N	N	Tim Tam Classic	N
**Bluebird**	N	N	Originals Ready Salted Chips	N
**Cadbury**	N	N	Dairy Milk	N
**Eta**	N	N	Ready Salted Ripples	N
**Griffin’s**	N	N	Mallowpuffs Original	N
**Flora**	Y	Y	Flora Original	Y
**Fresh’n Fruity**	Y	N	Mixed Berry Yoghurt Six Pack	Y
**Mainland**	N	N	Grated Tasty Cheese	N
**Maggi**	N	N	Rich and Saucy Beef Casserole	N
**McCain**	Y	N	Pub Size Chicken Kiev	N
**Meadow Fresh**	Y	N	Original Milk	Y
**Meadow Lea**	Y	Y	Original Margarine	Y
**Quality Bakers**	Y	Y	Muffin Splits	Y
**Sanitarium** ^**1**^	N	N	Weetbix	Y
**Sealord**	Y	Y	New Zealand Hoki Fish Fingers Classic Crumb	Y
**Tegel** ^**1**^	Y	Y	Memphis BBQ Wraps	N
**Tip Top**	N	N	Pineapple Fruju	N
**Wattie’s**	Y	N	Spaghetti in Tomato Sauce	Y
**Whittaker’s**	N	N	Creamy Milk	N

^1^ *Highlighted rows indicate different result for both thresholds tested and the WHO CLICK method*.

Comparing the selected thresholds to the WHO method for packaged foods, there was an 85% agreement for the 50% threshold and 65% agreement for the 75% threshold. Only three brands had different categorisations with the 50% threshold: McCain, Sanitarium and Tegel. Sanitarium is an example of where this method may be unsuitable for accurately classifying brands. The first visible product on the Sanitarium website, and likely a top-selling product, was Weetbix. Weetbix meets the criteria for being permitted to be marketed to children, but does not reflect the overall range of products sold by Sanitarium. The brand also sells a wide range of cereals, plant-based milks and breakfast drinks. Only 23% of the product range was permitted to be marketed to children under the WHO WPRO NPM largely due to added sugars or total sugar content. Conversely, based on the WHO CLICK method, chicken producer Tegel would not be able to market despite meeting both the 50% and 75% thresholds. The most visible product was Memphis BBQ wraps, which failed the NPM due to elevated sodium content.

For beverage brands, using the WHO CLICK method for the most visible product with the selected brands appeared to be a reasonably accurate reflection of a brand’s overall product selection. There was 100% agreement between beverage brands for the 50% threshold, 75% threshold and WHO CLICK method.

## Discussion

A preliminary literature review did not identify any published studies that utilised the WHO CLICK method to classify food and beverage brands, though publications may be forthcoming. A variety of other methods have been mentioned in the literature to classify brands including expert surveys [6,28,31], defining these by ‘brand category’ or best-known product(s) [16,27,32,33], and varying methods of ‘overall’ nutritional profile assessments of a brand [7,12,23,25,26,30,34,39,40]. Overall, however, there is limited detail provided on the use of these methods to classify a food or beverage brand. Furthermore, several studies identify that brand-only marketing is common, however choose to exclude this from analysis [3,41–44]. It is possible that monitoring of brand-only marketing is limited due to the lack of a widely used and tested method for classification.

A threshold model was demonstrated to be a feasible and robust method for classifying the healthiness of a food or beverage brand, and whether it should be permitted to be marketed to children. The thresholds tested provided a range of levels of restrictiveness. Interestingly, even the lowest threshold tested still restricts most brands that might be considered unhealthy, including the majority of fast-food brands and all beverage brands with products with high sugar content. The 50% and 75% thresholds are likely to be most feasible in a policy setting as they restrict all brands selling a majority of products that are not permitted. The 90% threshold was highly restrictive and would provide significant protection for children against marketing of most food brands. However, critics may claim this is more restrictive than necessary to achieve the desired effect. The high proportion of permitted products required could open the door to industry challenges [24].

Comparison of the WHO CLICK method to the 50% and 75% thresholds highlighted that while most brands could potentially be classified by the most visible product accurately, this is not universally the case. Most notably, Sanitarium has only 23% of products able to be marketed to children, however based on the WHO CLICK method they would be permitted to be marketed to children. Several brands had <50% of products permitted, though could make use of a loophole in the WHO CLICK method if they placed a permitted product as most visible on the website in order to be able to promote the brand. Additionally, use of website marketing may not be reflective of what product has the most visible marketing for a brand overall or in all media. Other mediums such as YouTube, and social media applications (e.g. Instagram, TikTok and Snapchat) may be more prominent in marketing to children. If industry were aware that this method was widely used, they may simply choose to position a healthier product option at the top of their website without changing overall marketing strategy.

The threshold method (when >50%) has been demonstrated to be a feasible and robust tool for classifying a brand, and it is the most rigorous method currently available for classifying a brand as healthy or unhealthy. This is an objective assessment of a brand’s product line, which has been shown to restrict brands whose products may harm children’s health. Use of a 75% threshold should also be considered to provide a more restrictive method. However, given that no dairy product brands are able to market at that threshold and these products are currently recommended for everyday consumption by children in NZ [45], the WHO WPRO model analysis may need to be considered alongside national dietary guidelines. Alternatively, a NPM that permits dairy products recommended for consumption by children in NZ could be trialled, for example with the condition of yoghurts having no added sugars. Such restrictions may seem unrealistic in a dietary guideline sense, however this would not stop brands from selling these products for consumption by children, it would simply limit the ability for marketing in ways that appeal to children, and/or in media and settings where children are a significant audience.

Comparison of the WHO method to a threshold method highlighted that while most brands could be accurately categorised by the most visible product, there were some important exceptions. The threshold method requires a country-specific nutrient database of products to provide a rigorous assessment of the brand healthiness. Developments in using artificial intelligence to web-scrape this data would make this option more feasible in the future. A key consideration is that methods that are suitable in a research setting for monitoring may be ineffective for policy purposes. Policy makers may require methods that are more practical with fewer resources required to allow for ongoing monitoring to be completed. Such methods may be appealing in a policy setting also to allow for straightforward monitoring and enforcement of regulations. However, any methods used that underpin regulation of unhealthy products are likely to come under scrutiny from the industry [24], and must be robust as well as comprehensive to minimise potential loopholes which could allow ongoing harmful brand marketing.

### Contribution to knowledge and implications for further research

To the authors’ knowledge, this is the first study that has primarily explored a method for classifying a food or beverage brand as healthy or unhealthy, nor are there any published reviews in the literature on methods available for classifying a brand. This study contributes to policy and practice by proposing and piloting a robust threshold method, and demonstrating that this can be successfully carried out given the necessary nutrient composition data is available. Further, the comparison of a threshold method to the WHO method has highlighted that while use of a ‘top’ product is largely aligned with thresholds, there may be critical inconsistencies within the sample of brands. This study of varying thresholds provides an alternative, rigorously tested approach to classifying a brand which is arguably less arbitrary than the WHO CLICK method.

Avenues for future research include considering the feasibility of using the threshold method in a policy setting to restrict brand marketing to children. A qualitative study may be beneficial to assess stakeholders’ opinions of a threshold model and which threshold should be used in practice. Further research could also look at a wider range of brands, particularly focusing on those that may be most likely to market to children or have a large proportion of products that may be consumed by children i.e., snack or cereal brands. Finally, it would be beneficial to assess how many of the brands that participate in sponsorship or other brand-only marketing in NZ, particularly those to which children are exposed, would be not permitted by use of an agreed threshold.

### Strengths and limitations

The threshold method provides an objective measure of a brand. It requires minimal interpretation or subjective decision making. The overall nutrient profile of a brand is captured with this model. Therefore, brands may be less likely to challenge the outcome as it is based on all their available products. The decision of which threshold to use may be more likely to be contested by industry and therefore may require further consultation and testing to ensure the method is as robust as possible.

Further strengths include the use of the WHO WPRO NPM for the assessment of thresholds, as it is specifically designed for monitoring marketing to children and is also specific to the Western Pacific Region [38]. The model is straightforward to use and freely available, ensuring that the study is easily reproducible. Additionally, thanks to the Nutritrack database, a wide range of product categories were able to be assessed from the selected brands for packaged foods.

There were some key limitations, primarily surrounding the availability of data. The method requires a high quality existing nutrition database, such as the Nutritrack database used for this study. Such databases are unlikely to be universally accessible, particularly in low- and middle-income countries. The fast-food database had a large proportion of nutrient information missing with 58% of products having no nutrient information. The percentage of missing data varied between fast food brands. This meant that a comprehensive assessment of fast-food brands was not possible, and the proportion of products permitted to be marketed to children will vary in accuracy between brands.

## Conclusion

Development of mandatory legal frameworks for brand-only advertising are essential to upholding children’s rights. The threshold method demonstrated in this study remains the most rigorously tested option for classifying a brand as ‘healthy or unhealthy’, or permitted or not permitted to be marketed to children. It is also feasible and relatively straightforward once the nutrient profile method is understood. It is therefore recommended that a threshold greater than 50% should be used to ensure that children are only exposed to marketing for brands where the majority of foods are considered healthy. While there will be avenues for brands to create loopholes, regular review of any method would be required in a policy or research setting to adapt to the ever-changing food marketing environment.

## Supporting information

S1 Table Total number of products analysed and permitted to be marketed to children by packaged food brand with major categories. (DOCX)

S2 Table Total number of products analysed and permitted to be marketed to children by packaged drinks brand. (DOCX)

S3 Table Total number of products analysed and permitted to be marketed to children by fast food brand (excluding sauces). (DOCX)
