# Supplementary material for: Testing a nutrient composition threshold model to classify brands for marketing restrictions
Source: PLoS One. 2024 Oct 25;19(10):e0311579. doi: 10.1371/journal.pone.0311579 (PMC11508487; doi:10.1371/journal.pone.0311579)
Supplement: S1 Table — (DOCX) [file pone.0311579.s001.docx]

**Table S1: Total number of products analysed and permitted to be marketed to children by packaged food brand with major categories.**

| **Packaged food brand** | **Number of products** | **Number**  **permitted** | **Percentage permitted** | **Major Categories** |
| --- | --- | --- | --- | --- |
| Anchor | 100 | 50 | 50% | Milk, Yoghurts, Cheese |
| Arnott’s | 91 | 0 | 0% | Cakes/Sweets, Savoury snacks |
| Bluebird | 35 | 0 | 0% | Savoury snacks |
| Cadbury | 116 | 0 | 0% | Chocolate and confectionary |
| Eta | 73 | 0 | 0% | Savoury snacks, Sauces |
| Flora | 10 | 10 | 100% | Butter and other fats and oils |
| Fresh’n Fruity | 14 | 7 | 50% | Yoghurts |
| Griffin’s | 60 | 0 | 0% | Cakes/Sweets, Savoury snacks |
| Maggi | 113 | 40 | 35% | Ready-made meals, Sauces |
| Mainland | 66 | 0 | 0% | Cheese, Butter |
| McCain | 61 | 44 | 72% | Readymade meals, Frozen fruit/vegetables |
| Meadow Fresh | 104 | 62 | 60% | Milk, Yoghurts, Cheese |
| Meadow Lea | 7 | 7 | 100% | Butter and other fats and oils |
| Quality Bakers | 10 | 8 | 80% | Bread and bread products |
| Sanitarium | 96 | 22 | 23% | Milk, Cereals |
| Sealord | 84 | 82 | 98% | Fresh and frozen fish,  Processed fish |
| Tegel | 78 | 64 | 82% | Fresh and frozen poultry, Processed poultry |
| Tip Top | 52 | 0 | 0% | Edible ices |
| Wattie’s | 306 | 176 | 58% | Readymade meals, Fresh and frozen fruit/vegetables, Processed fruit and vegetables, Sauces |
| Whittaker’s | 69 | 0 | 0% | Chocolate and confectionary |
