# Supplementary material for: Testing a nutrient composition threshold model to classify brands for marketing restrictions
Source: PLoS One. 2024 Oct 25;19(10):e0311579. doi: 10.1371/journal.pone.0311579 (PMC11508487; doi:10.1371/journal.pone.0311579)
Supplement: S2 Table — (DOCX) [file pone.0311579.s002.docx]

**Table S2: Total number of products analysed and permitted to be marketed to children by packaged drinks brand.**

| **Packaged drinks brand** | **Number of products** | **Number permitted** | **Percentage permitted** |
| --- | --- | --- | --- |
| Charlie’s | 29 | 1 | 3% |
| Coca-Cola | 51 | 0 | 0% |
| E2 | 7 | 0 | 0% |
| Fresh Up | 15 | 0 | 0% |
| H2Go | 5 | 0 | 0% |
| Just Juice | 22 | 3 | 12% |
| Keri Juice Co | 40 | 0 | 0% |
| Kiwi Blue | 8 | 6 | 75% |
| L&P | 10 | 0 | 0% |
| McCoy | 11 | 1 | 9% |
| Mizone | 4 | 0 | 0% |
| NZ Natural | 5 | 0 | 100% |
| Powerade | 8 | 0 | 0% |
| Pump | 2 | 2 | 100% |
| Pure Dew | 1 | 1 | 100% |
| Pure NZ | 1 | 1 | 100% |
| Red Bull | 15 | 0 | 0% |
| Schweppes | 41 | 3 | 7% |
| Sprite | 14 | 0 | 0% |
| V | 36 | 0 | 0% |
