# Supplementary material for: Testing a nutrient composition threshold model to classify brands for marketing restrictions
Source: PLoS One. 2024 Oct 25;19(10):e0311579. doi: 10.1371/journal.pone.0311579 (PMC11508487; doi:10.1371/journal.pone.0311579)
Supplement: S3 Table — (DOCX) [file pone.0311579.s003.docx]

**Table S3: Total number of products analysed and permitted to be marketed to children by fast food brand (excluding sauces).**

| **Fast-food brand** | **Number of products** | **Number permitted** | **Percentage permitted** |
| --- | --- | --- | --- |
| Burger Fuel | 35 | 5 | 14% |
| Burger King | 140 | 6 | 4% |
| Domino’s Pizza | 125 | 2 | 2% |
| Hell Pizza | 98 | 24 | 24% |
| KFC | 56 | 12 | 21% |
| McDonald’s | 265 | 11 | 4% |
| Pita Pit | 30 | 5 | 17% |
| Pizza Hut | 309 | 76 | 25% |
| St Pierre’s Sushi | 82 | 57 | 70% |
| Subway | 76 | 40 | 53% |
| Tank Juice | 42 | 12 | 29% |
| Wendy’s | 99 | 18 | 18% |
